# Erosive tooth wear – knowledge and treatment preferences among dental hygienists

**DOI:** 10.2340/aos.v85.46261

**Published:** 2026-06-11

**Authors:** Julie Marie Haabeth Brox, Amela Tulek, Qalbi Khan, Amer Sehic, Linda Stein, Anne Breivik, Tor Paaske Utheim, Aida Mulic

**Affiliations:** a Department of Oral Biology, Faculty of Dentistry, University of Oslo, Oslo, Norway; b Nordic Institute of Dental Materials (NIOM AS), Oslo, Norway; c Department of Public Health and Sport Sciences, Inland Norway University of Applied Sciences, Elverum, Norway; d Department of Maxillofacial Surgery, Oslo University Hospital, Oslo, Norway; e Department of Clinical Dentistry, Faculty of Health Sciences, UiT The Arctic University of Norway, Tromsø, Norway; f Department of Medical Biochemistry, Oslo University Hospital, Oslo, Norway; g Department of Plastic and Reconstructive Surgery, Oslo University Hospital, Oslo, Norway; h Department of Ophthalmology, Sørlandet Hospital Trust, Arendal, Norway

**Keywords:** Erosive tooth wear, preventive measures, dental hygienists, young adults

## Abstract

**Objective:**

Erosive tooth wear (ETW) is generally recognized as a widespread condition and a rising concern within the oral healthcare system. This study aimed to investigate dental hygienists` experiences and treatment methods regarding ETW in young adults.

**Material and methods:**

A questionnaire was sent to 1,084 Norwegian dental hygienists working in both public and private dental services. In total, 313 responded (response rate 28.9%). After excluding 47 respondents who did not meet the inclusion criteria of working with young adults (15–30 years), 266 dental hygienists were included in the final analyses (24.5% of those invited).

**Results:**

In total, 96% of the study population of 266 dental hygienists reported recording ETW in the register, with the public dental service doing so more frequently than the private sector. Sports drinks, energy drinks and soft drinks were believed to be the primary risk indicators of ETW, analogously across groups. Furthermore, there was consensus that the most frequently performed preventive measures were dietary guidance and self-administration of fluoride rinse and/or tablets.

**Conclusions:**

Overall, this study suggests that, in our sample, dental hygienists possess substantial knowledge of ETW and hold strong views on potential improvements in their work as healthcare professionals.

## Introduction

In recent years, the shift towards a modern way of living, with frequent consumption of acidic drinks and food, has raised a deep concern regarding erosive tooth wear (ETW), within the oral healthcare system. ETW is tooth wear in which dental erosion is the primary etiological factor. Dental erosion is the chemical loss of mineralized tooth substance caused by exposure to acids not derived from oral bacteria [1]. Worldwide, an estimation of the prevalence of ETW in permanent teeth is reported to be 20%–45% [2]. In Norway, three main research studies have described the prevalence of ETW among young adults. In 2013, the prevalence of ETW among 18-year-olds was 38%, in Oslo. In 2016, a similar percentage was determined among 16-year-olds in Tromsø, while in the western part of Norway, the prevalence was in 2014 estimated to be 59% among 16–18 year olds [3–5]. ETW can lead to serious consequences, such as imbalanced occlusion with functional loss, aesthetic alterations and pain [6]. In situations involving dentin and dentinal tubules, this can cause hypersensitivity, which may affect the patient’s quality of life, with tenderness and discomfort [7, 8]. In case of masticatory dysfunction or sensitive teeth, this can compromise the capacity of drinking or eating, and with time lead to malnutrition [9].

Numerous preventive measures can be implemented to preserve the dentition and/or halt the progression of ETW. Various fluoride treatments are included in the non-operative approach, following the minimal invasive strategy. Without proper preventive strategies, active lesions will continue to worsen [10]. In critical cases, operative treatment can be challenging, expensive, and invasive [11, 12]. In Norway, in cases of severe ETW, that is, affecting oral function and aesthetics, treatment is provided with refund from the Norwegian government [13]. In 2023, the cost for the treatment of ETW was estimated at approximately 287 million Norwegian kroner (≈ 24.7 million euros), representing 10.3% of the total reimbursements that year [14]. This suggests that ETW is not only an individual problem, but also a significant economic burden for society. Thus, primary prevention ought to be prioritized, that is, to prevent disease or injury before it occurs [15].

An essential task for dental health personnel is to recognize, restrain, or phase out the susceptibility to acidic environments. Dental hygienists have a unique role in oral health promotion [16]. In Norway, dental hygienists complete a 3-year bachelor’s degree (180 European Credit Transfer and Accumulation System [ECTS] credits) that combines theoretical and clinical training in oral health promotion, disease prevention, examination, and non-invasive treatment [17]. They are authorized to perform oral examinations, take radiographs, and diagnose common oral diseases within their competence. Dental hygienists may make independent diagnostic and treatment decisions for conditions within their scope of practice, but complex cases should be referred to a dentist [18]. In contrast, the dentists are often more preoccupied with operative treatment [19]. In Norway, the Dental Health Service Act state that the public dental services are responsible for the endorsement of preventive oral health, public information and necessary treatment [20]. In the Norwegian dental services, approximately 9% of all employees are dental hygienists, covering 20 per 10,000 inhabitants nationally [21].

In Norway, the comprehension of dental hygienists’ experiences and treatment methods regarding ETW among young adults is sparse. Thus, the current study aimed to obtain new insights of the challenges faced by dental hygienists regarding ETW. A secondary objective of this study was to investigate the variations between public and private dental health services and their approaches to ETW.

## Methods

### Study design and population

**Figure 1:**
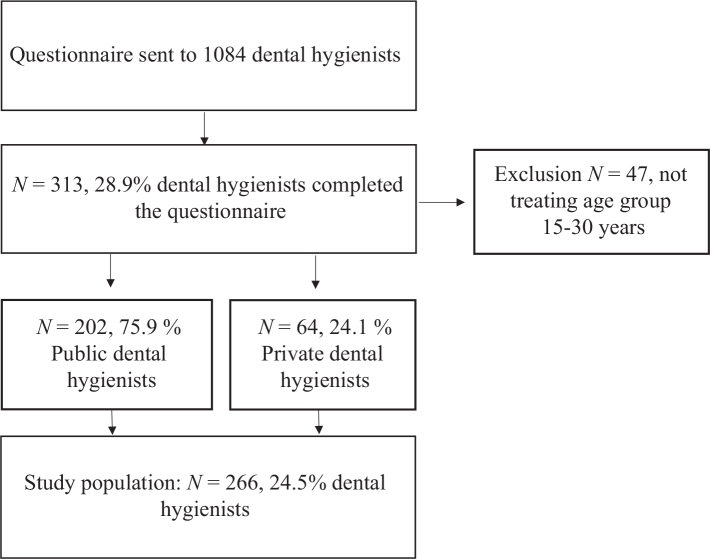
Study population described by a flowchart.

The present study was performed as a partial precoded questionnaire, including some possible descriptive responses in text. The questionnaire was addressed electronically to all dental hygienists (*n* = 1,084) registered as members of The Norwegian Dental Hygienist Federation (NTpF) in December 2024. All regions in Norway were represented (Table 1). The questionnaire was designed using the Internet-based software program ‘Nettskjema’, developed by the University of Oslo. The questionnaire was refined through multiple iterative rounds of expert panel review to ensure clarity and content validity. This study included dental hygienists both working in public and private sectors. A trial run of the questionnaire was carried out by private and public dental hygienists (*n* = 6), with broad experience, prior to conducting the study. Feedback highlighted a few questions that could be rephrased or measured differently, while confirming that the questionnaire was generally clear and functioned well. This process contributed to the overall reliability of the questionnaire. Five reminders were sent to encourage responses to the questionnaire, two by SMS and three by e-mail. The questionnaire was sent to the members of NTpF from December 2024 until April 2025. Of 1,084 dental hygienists invited to participate, 313 replied to the questionnaire, giving a response rate of 28.9%. However, 47 participants were excluded due to not working clinically with young adults (15–30 years). Hence, the study population consisted of 266 dental hygienists, resulting in a response rate of 24.5% (Figure 1).

**Table 1:** Study population (*n* = 266).

Clinical management of patients; 15–30 years	Public dental service 75.9 % (*n* = 202) % (*n*)	Private dental service 24.1 % (*n* = 64) % (*n*)	Total (*n* = 266) % (*n*)
**Workplace divided by regions**			
Northern Norway	16.3 (33)	9.4 (6)	14.7 (39)
Central Norway	7.9 (16)	17.2 (11)	10.2 (27)
Western Norway	24.7 (50)	29.7 (19)	25.9 (69)
Southern Norway	4.6 (10)	1.6 (1)	4.1 (11)
Eastern Norway	46.0 (93)	42.2 (27)	45.1 (120)
**Employed population by place of work**			
200–1,999	2.0 (4)	0	1.5 (4)
2,000–19,999	40.6 (82)	31.2 (20)	38.3 (102)
20,000–99,999	43.1 (87)	40.1 (26)	42.5 (113)
100,000–≥ 100 000	14.4 (29)	28.1 (18)	17.7 (47)
**Graduation year**			
1970–1989	8.9 (18)	1.6 (1)	7.1 (19)
1990–1999	13.9 (28)	16.4 (10)	14.3 (38)
2000–2009	24.8 (50)	20.3 (13)	23.7 (63)
2010–2024	52.5 (106)	62.5 (40)	54.9 (146)

### Questionnaire

The questionnaire was grounded on an evaluation of relevant literature [3, 5, 22–25]. This questionnaire included two parts. The first part gathered information about work location according to regions, approximate number of inhabitants, year of graduation, working in public or private dental service and if they provided treatment to patients between the ages of 15 and 30. If responders were not treating this age group, they were guided to the last page of the questionnaire. The second part aimed to explore methods of diagnostic recording in the register, assumed risk indicators, performed preventive treatment and opportunities of enhancement considering ETW. Dental hygienists learn Visual Erosion Dental Examination (VEDE) scoring system during their education [26]. The VEDE system consists of five grades, and students are educated to classify ETW according to these levels. As part of their education, they use a laminated guide that contains clinical photographs of each VEDE grade. This reference sheet is commonly found in dental clinics and often kept at each dental unit. It helps ensure consistent scoring and calibration. In this questionnaire, most of the questions were mandatory. The questionnaire included three open-text fields to express individual opinions. For instance: ‘Why are you not recording ETW in the register?’.

### Statistical analyses

Descriptive analysis was carried out to describe the participants work routines, opinions and challenges. Chi- square test was used to test for differences between public and private health services regarding the treatment of ETW in young adults. The significance level was assigned to a *p*-value of *p* < 0.05. These analyses were conducted using the statistical software package Stata/SE 18.0. In addition, multivariable logistic regression analysis was performed with ETW as the dependent variable and municipality population size and year of graduation as independent variables. Due to the limited number of events in the smaller outcome group (*n* = 10), the number of predictors was restricted to reduce the risk of overfitting and unstable estimates. All statistical analyses were conducted using R (R Foundation for Statistical Computing, Vienna, Austria). The study is reported in accordance with the STROBE guidelines for cross-sectional studies.

### Ethical considerations

The current study was approved by Sikt (The Service Provider for the Knowledge Sector, id 652369). The Norwegian Dental Hygienist Federation accepted the survey and contributed with the dissemination of the questionnaire by emailing them with high confidentiality to members. The online survey program ‘Nettskjema’ provided complete anonymity of the participants.

## Results

Throughout Norway, dental hygienists from both public (*n* = 202) and private dental services (*n* = 64) participated in this questionnaire, as described in Table 1. The findings showed that dental hygienists in the public dental services worked significantly more frequently with young adults (81.2% daily), than those working in private dental services (21.3% daily). Hygienists in the public services affirmed to register ETW in the register and operated with a procedure more accurate than a two-levelled system (enamel/dentin), significantly more often than private dental hygienists. The most severe ETW was observed on maxillary palatine surfaces and mandibular occlusal surfaces. Public dental hygienists observe patients with ETW more frequently; they usually find the ethology and perform a nutritional assessment significantly more often than private dental hygienists (Table 2).

**Table 2:** Distribution of the following treatment strategies in patients aged 15–30 years.

Variables	Public dental service (*n* = 202)	Private dental service (*n* = 64)	*P*
**Frequency of treatment (*n* = 266)**			
Daily	81.2 (164)	21.3 (13)	**< 0.001**
Several time per week	15.4 (31)	43.8 (28)	
Occasionally	3.5 (7)	35.9 (23)	
Seldom/never	0	0	
**Do you register ETW in the journal (*n* = 266)**			
Yes	97.5 (197)	90.6 (58)	**0.016**
No	2.5 (5)	9.4 (6)	
**If you register ETW, do you utilize a specific system (*n* = 260)**			
No	35.2 (70)	75.4 (46)	**< 0.001**
Yes, I separate enamel and dentin lesions (two-levelled system)	17.6 (35)	16.4 (10)	
Yes, a system more detailed than two levelled	44.2 (88)	4.9 (3)	
Other specified in text	3.0 (6)	3.3 (2)	
**How do you register ETW in the journal (*n* = 261)**			
Symbol/number on each tooth surface	64.5 (129)	65.6 (40)	0.560
Symbol/number each tooth with ETW	14.0 (28)	14.8 (9)	
Symbol/number in total, patient level	0.5 (1)	1.6 (1)	
Description in text	14.5 (29)	16.4 (10)	
Other	6.5 (13)	1.6 (1)	
**Why do you not register ETW (*n* = 44)**			
Uncertain how to register	6.9 (2)	20.0 (3)	0.229
The journal lacks an appropriate section for recording this information	34.5 (10)	20.0 (3)	
I think ETW are difficult to treat	17.0 (5)	6.6 (1)	
I don’t have time	24.1 (7)	13.3 (2)	
Other, specified	17.2 (5)	40.0 (6)	
**Where do you find the most severe ETW (*n* = 266)**			
***Buccal surface maxilla***			
Severe	21.3 (43)	25.0 (14)	0.707
Moderate	16.8 (34)	12.5 (8)	
Minor	61.9 (125)	65.6 (42)	
***Palatal surface maxilla***			
Severe	55.9 (113)	45.3 (29)	0.151
Moderate	23.8 (48)	35.9 (23)	
Minor	20.3 (41)	18.7 (12)	
***Occlusal surface maxilla***			
Severe	26.2 (53)	18.8 (12)	0.137
Moderate	42.1 (85)	56.3 (36)	
Minor	31.7 (64)	25.0 (16)	
***Buccal surface mandibula***			
Severe	18.8 (38)	23.4 (15)	0.689
Moderate	13.4 (27)	14.1 (9)	
Minor	67.8 (137)	62.5 (40)	
***Lingual surface mandibula***			
Severe	19.3 (39)	20.3 (13)	0.971
Moderate	18.3 (37)	17.2 (11)	
Minor	62.4 (126)	62.5 (40)	
***Occlusal surface mandibula***			
Severe	64.9 (131)	57.8 (37)	0.173
Moderate	12.4 (25)	17.2 (14)	
Minor	22.8 (46)	20.3 (13)	
**Do you see ETW most frequent in (*n* = 266)**			
Female	0.5 (1)	1.6 (1)	
Men	37.6 (76)	28.1 (18)	0.284
No difference	61.9 (125)	70.3 (45)	
**Do you see a higher number of patients with ETW today, than before (*n* = 266)**			
No	10.9 (22)	37.5 (24)	**< 0.001**
Yes	89.1 (180)	62.5 (40)	
**Normally, do you find the aetiology of ETW in patients (*n* = 266)**			
Always	84.7 (171)	70.3 (45)	**0.017**
Occasionally	14.4 (29)	29.7 (19)	
Rarely	1 (2)	0	
**Do you include a dietary analysis for patients with ETW (*n* = 264)**			
Always	30.0 (60)	14.0 (9)	**0.040**
Occasionally	24.0 (48)	28.1 (18)	
Never	46.0 (92)	57.8 (37)	

ETW: erosive tooth wear.

**Figure 2:**
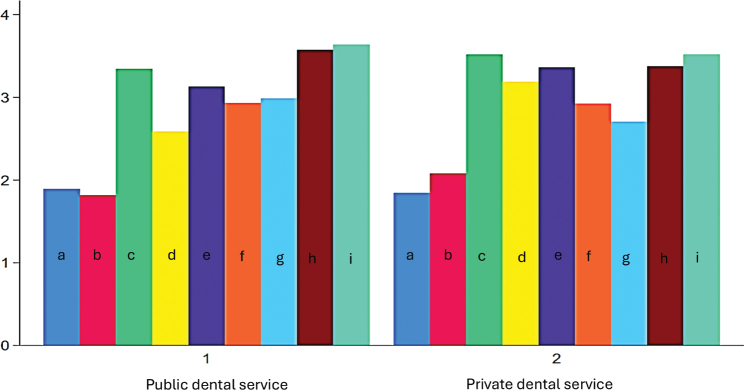
Risk indicators for ETW. The y-axis represents mean level (1–5), where level 1 is considered the most probable aetiology of ETW. None of the values reach level 5; therefore, only levels 1–4 are displayed in the graph. The x-axis represents the variables a–i, divided into two groups. Group 1: Public Dental Service, group 2: Private dental service. Variables represent a: soft drinks, b: sports drinks/energy drinks, c: water with taste, d: fruit juice, e: citrus fruit, f: acidic food and sweets, g: GER(D) and eating disorders, h: hyposalivation, i: medications.

**Figure 3:**
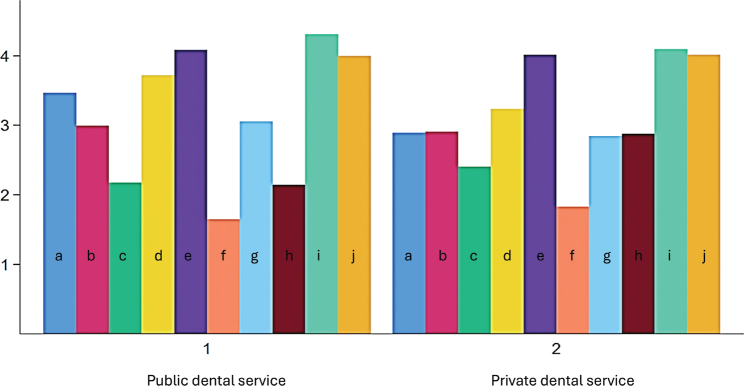
Preventive measures of ETW performed by dental hygienists. The y-axis represents mean level (1–5), and level 1 is the most common treatment method. None of the values reach level 5; therefore, only levels 1–4 are displayed in the graph. The x-axis describes the variables (a–j), divided into two groups. Group 1: Public dental service and Group 2: Private dental service. Variables represent a: Duraphat toothpaste, b: toothpaste against ETW, c: fluoride rinse or tablets, d: products aimed to reduce hyposalivation, e: chlorhexidine rinse, f: guidance to change diet, g: fluor protector, h: fluoride varnish, i: SDF (silver diamine fluoride), j: no treatment.

The results showed a broad consensus between groups that soft drinks, sports drinks, and energy drinks are among the key risk indicators for developing ETW. Subsequently, fruit juice, gastroesophageal reflux disease (GERD), and eating disorders were also reported as primary indicators associated with the onset and progression of ETW (Figure 2). A majority of the dental hygienists agreed that key elements of preventive treatment are use of fluoride rinse or tablets at home, and a guidance to a change of nutrients. Then follows, fluor protector or fluoride varnish applied in clinic (Figure 3). Thus, both groups acknowledged ETW as a community concern (Table 3). Dental hygienists in both public and private services appeared to share an understanding of their main tasks to prevent and stall the progression of ETW. They communicated a desire to gain a better understanding of possible diagnostic and treatment methods with new approaches better provide for the young generation. Additionally, several dental hygienists proclaimed marketing strategies of soft drinks as a key factor contributing to the problem. Hence, both groups believe in improvement of parental awareness and the undesirable consequences of ETW (Table 3).

**Table 3:** Knowledge and awareness of ETW among dental hygienists.

Do you agree or disagree with the following statements (*n* = 266)	Public dental service 75.9 % (*n* = 202) % (*n*)	Private dental service 24.1 % (*n* = 64) % (*n*)	*P*
I believe ETW is a societal problem	96.5 (195)	86.0 (58)	0.056
I believe advertisement targeting young people should be stricter	99.5 (201)	96.9 (62)	0.083
The need for higher parental consciousness about the negative effects of energy drinks/soft drinks	98.5 (199)	98.4 (63)	0.965
Patients change risk habits according to advice and guidance	67.8 (137)	75.0 (48)	0.277
I find it challenging to prevent ETW	81.7 (165)	73.4 (47)	0.153
My education on ETW has been very limited	39.1 (79)	43.8 (28)	0.509
I would like to gain more knowledge about the diagnostic and treatment methods for ETW	84.2 (170)	79.7 (51)	0.406
My workday is busy, and I don’t have time to focus on ETW	37.6 (76)	26.6 (17)	0.106
There is a need for national guidelines for dental hygienists regarding the diagnosis and treatment of ETW	91.1 (184)	85.9 (55)	0.234

ETW: erosive tooth wear.

In the open-ended question regarding utilization of specific ETW registration system, 7.5% of dental hygienists reported the use of either VEDE scoring system or their own method describing in text, numbers or symbols [26]. In the final page of the questionnaire, where the participants could share their opinions, 3% of dental hygienists expressed a desire to improve the current registration method of ETW in the register and requested that it be structured more like the dental caries registration. Three percent of the participants emphasized the importance of greater political engagement in controlling the marketing, sale, and pricing of acidic beverages. As a final point, 5.3% of dental hygienists reported being worried about the lack of health literacy in the entire population and suggested public posters and information campaigns (data not presented).

In the multivariable logistic regression analysis, municipality population size was significantly correlated with ETW recording (*p* = 0.047). Dental hygienists working in municipalities with ≥100,000 inhabitants had lower odds of recording ETW compared with those in municipalities with < 20,000 inhabitants (odds ratio [OR] = 0.09, 95% confidence interval [CI]: 0.00–0.65) (Table 4). Also, graduation year was significantly associated with ETW recording (*p* = 0.013). Dental hygienists graduating between 2010 and 2024 had lower odds of recording ETW compared with those graduating before 2010 (OR = 0.12, 95% CI: 0.01–0.68) (Table 4).

**Table 4:** Factors associated with ETW recording.

Variable	OR	95% CI	*p*-value
Municipality population size			**0.047**
< 20,000	**-**	**-**	
20,000–99,999	0.19	0.01, 1.23	
100,000+	0.09	0.00, 0.65	
Graduation year			**0.013**
Pre-2010	**-**	**-**	
2010–2024	0.12	0.01, 0.68	

CI: confidence interval; OR: odds ratio.

Bold values indicate statistically significant differences (p < 0.05)

## Discussion

This study aimed to explore dental hygienists’ experiences and treatment methods regarding ETW among young adults. The purpose was also to assess whether there was a difference between public and private dental hygienists’ approach to ETW. Dental hygienists have a significant role through educational guidance of oral health behaviour, engagement strategies, and disease prevention [16]. In the multivariable analyses, the results indicating that smaller municipalities register ETW more often could indicate closer patient follow-up or differing organizational practices. Also, the finding that participants who graduated before 2010 were more likely to record ETW may reflect increased clinical experience or differences in educational emphasis. Although statistically significant, the wide confidence intervals indicate considerable uncertainty around the estimates, likely due to the small sample size in one of the outcome groups. According to the findings in the Chi-square analysis, there was a significant difference between public and private dental service in the frequency of treating patients aged 15–30 years. Public hygienists are treating patients in this age group more frequently than private hygienists. This is mostly due to the organization structure of the Norwegian dental service, where public dental service is founded by the government and delivers oral health service to those given priority in The Dental Health Service Act. That includes children, young adults and the aging population in institutional or in-home care [20]. In contrast, the private dental hygienists treat the remaining part of the adult population that primarily pay out of pocket for dental treatment [27]. The exception is patients with rare diseases or vulnerabilities receiving treatment coverage by the reimbursement policies [13]. Similar observations are reported within other Nordic countries, where private and public dental hygienists carry out various, role-specific tasks [28–30]. Subsequently, there was a discrepancy between the frequency of registration of ETW, between groups. Public dental hygienists registered ETW significantly more often and utilized a registration system more detailed than a two-levelled system in the register than private dental hygienists. Hence, to record the onset and progression of ETW, the assistance of an accurate grading system is of the essence [31]. In the free text option, VEDE [26] was the only grading system that was mentioned. Additionally, some dental hygienists reported being troubled by the grading system, together with the absence of an appropriate register system to register in. Furthermore, by comparing current results, that is, the recording of ETW with previous questionnaires among dentists, the registration rates indicate strong similarities [24, 32]. To better identify and to assess changes over time, it may be beneficial to implement new tools for detection and monitoring of ETW. To the best of our knowledge, intraoral scanner-based monitoring of ETW is rarely used by dental hygienists, possibly due to economic constraints, insufficient institutional support, limited training and time pressure. However, digital scanning technology represents a promising future tool that could be more widely integrated into dental hygiene education and clinical practice [33–35]. These tools allow, among other things, high precision and sensitivity, comparison over time, and good documentation of ETW [36]. The development of AI-based diagnostic approaches could represent a future direction for improving the detection and assessment of ETW [35]. Clinically, in both private and public dental service, the most severe erosive lesions were found on maxillary palatal surfaces and mandibular occlusal surfaces. These results are also stated in previous studies [32, 37–39]. Nearly two thirds of dental hygienists thought ETW was evenly distributed between sexes, and roughly one third of dental hygienists considered males suffered from higher levels of ETW. Current findings, with a higher prevalence of ETW in men, are seen in similar studies [39–42]. There was a significant difference between groups regarding the tendency of observing higher ETW level today, than in the past. In this questionnaire almost nine out of 10 public dental hygienists reported finding increasing numbers of patients with ETW than previously. Stenhagen et al*.* [43] have suggested it to be the severity of ETW that has increased the last 30 years. However, Mulic et al. discovered the same perception among dentists in Norway and Island as in current survey, that is, higher prevalence of ETW today, compared with 10–15 years ago [24, 32].

Performing a dietary analysis is a valuable tool to identify the aetiology of ETW, that is, the behaviour and nutritional habits that are of essence in the pathogenesis [44, 45]. In this study, dental hygienists working in public practice discovered the erosive influencing factors, more frequently than those working in private practice. Thus, nearly one third of public dental hygienists responding to the study always performs a dietary analysis, significantly more often than private dental hygienists. Overall, nearly half of dental hygienists never carried out a dietary analysis. This results contrasts from the recommendations from National guidelines on oral health care, patients ought to be offered regularly dietary guidance in line with national dietary advices [46]. However, similar results have been recorded both by dental practitioners and dental hygienists [22, 32], which indicate a need for emphasis on dietary analysis in the future [1, 45].

Evenly across groups, dental hygienists acknowledged soft drinks, sports drinks and energy drinks as the key risk indicators of ETW. Subsequently, fruit juice and GERD were reported as risk indicators of ETW. Thus, in recent times, consumption of acidic drinks has grown substantially and is considered a major risk indicator of ETW [25, 47]. In Norway, analysis reveals that the most purchased item in grocery stores is soft drinks, preferred by consumers over cheese and milk [48]. Young adults are within the age group drinking the most acidic beverages [49]. Globally, Norway is rated in 8^th^ place based on soda consumption, giving almost 100 litres per person [50]. In agreement with our results, Järvinen et al*.* estimated a 37-time higher risk of evolving ETW in individuals consuming citrus fruit more than twice a day [51].

As regards preventive treatment of ETW, performed by dental hygienists, there is a consensus that guidance to a change of diet is the most important method, followed by at home usage of fluoride rinse or tablets. These findings are supported by the Consensus report of the European Federation of Conservative Dentistry, which states that evaluating all etiological factors is of major importance [52]. The current study suggests that Norwegian dental hygienists share similar views of risk indicators and preventive strategies targeting ETW.

In general, dental hygienists who responded to the questionnaire regarded ETW as a public health problem which is challenging to prohibit, partly due to intense market pressure and low parental awareness. However, Norway has recently introduced a broad national regulation banning the marketing of unhealthy beverages and food directed towards children under 18 years of age [53]. This regulation is in line with the WHO (World Health Organization) Global strategy and action plan on oral health, that calls for more upstream action with emphasis on the social and commercial determinants of oral health [54]. Such national regulations provide an improvement in preventive health measures intended to reduce diet-related health obstacles among the young generation in Norway. Current policy is thought to be under the influence from recent published health data by WHO that indicates that 24% of boys and 22% of girls in primary school are obese or overweight [55]. Additionally, high intake of soft drinks is recognized as a determining factor of the onset of diabetes and obesity, by this means a true challenge for overall health [56, 57]. As such, this regulation is also an example of measures aimed at addressing common risk factors.

In view of recent prevalence studies in young adults, dental hygienists’ preventive work is considered highly valuable [3, 41, 42, 58]. Accordingly, a project under development called ‘Care4YoungTeeth’, aims to motivate and guide young adolescents to develop healthy oral health routines, by creative and modern strategies, that is, games and movies [59]. Thus, various school districts strive to implement this project as a part of their educational program through active learning and visits from a public dental hygienist. This is one of many possible approaches to enhance the health literacy in the population. A recent analysis indicates low health literacy among 43% of the conscripts in Norway [60]. This finding implies that conscripts are not aware of the association between sugar and dental caries, or acidic drinks and ETW which raises great concern for future oral health. The novelty of this study lies in the consistent agreement among public and private dental hygienists on the need for upstream interventions to address ETW, as well as on the challenges of consistently recording ETW in patient records.

In this study, one limitation is the anonymously created design which reduced the scope of information about the non-responders. Furthermore, it is important to acknowledge that 28.9% is considered as a low response rate. In addition, some responders were excluded from the study, due to absence of clinical treatment of young adults, giving the inclusion rate of 24.5%. The questionnaire was developed from relevant literature, refined through expert panel review and tested in a small group of six experienced dental hygienists. However, formal validation was lacking, which may have introduced information bias. In addition, no standardized visual materials were provided to illustrate ETW, meaning that respondents relied on their own clinical experience, causing a potential bias through misclassification and variability in ETW scoring. Further limitations are similar to other questionnaires; the answers are grounded on the memory capacity among the responders [61]. In this study there are also some robust elements, the inclusion and/or exclusion criteria of treating the correct patient group, together with the large proportion of mandatory questions results in high accuracy of the data. Moreover, according to the incorporation of all regions in Norway from both public and private practice, this study should be considered as an insightful representation of the overall picture of oral healthcare service provided by dental hygienists in Norway.

## Conclusions

In conclusion, the study suggests that, in our sample, dental hygienists in Norway recognize and are motivated to address the rising problem of ETW among young adults. Public hygienists seem to perform a nutritional assessment and explore the ethology of ETW more frequently than private hygienists. Between groups of public and private dental services it appears to be a consensus for the main risk indicators of ETW, that is, soft drinks and sports drinks/energy drinks. The study suggests that, in our sample, dental hygienists recognize their roles and practices in managing ETW, while facing challenges in consistently recording ETW in patient records, and that some seem to express a desire for greater involvement from policymakers to implement upstream interventions. These findings should be interpreted in light of the study’s limitations, particularly the low response rate.

## Data Availability

The original contributions presented in the study are included in the article, and further inquiries can be directed at the corresponding author.

## References

[1] Schlueter N, Amaechi BT, Bartlett D, Buzalaf MAR, Carvalho TS, Ganss C, et al. Terminology of erosive tooth wear: consensus report of a workshop organized by the ORCA and the Cariology Research Group of the IADR. Caries Res. 2020;54(1):2–6. 10.1159/000503308 31610535

[2] Schlueter N, Luka B. Erosive tooth wear – a review on global prevalence and on its prevalence in risk groups. Br Dent J. 2018;224(5):364–70. 10.1038/sj.bdj.2018.167 29495027

[3] Mulic A, Tveit AB, Skaare AB. Prevalence and severity of dental erosive wear among a group of Norwegian 18-year-olds. Acta Odontol Scand. 2013;71(3–4):475–81. 10.3109/00016357.2012.696689 22762481

[4] Mulic A, Fredriksen O, Jacobsen ID, Tveit AB, Espelid I, Crossner CG. Dental erosion: prevalence and severity among 16-year-old adolescents in Troms, Norway. Eur J Paediatr Dent. 2016;17(3):197–201. 27759408

[5] Sovik JB, Tveit AB, Storesund T, Mulic A. Dental erosion: a widespread condition nowadays? A cross-sectional study among a group of adolescents in Norway. Acta Odontol Scand. 2014;72(7):523–9. 10.3109/00016357.2013.875588 24432788

[6] Schlueter N, Jaeggi T, Lussi A. Is dental erosion really a problem? Adv Dent Res. 2012;24(2):68–71. 10.1177/0022034512449836 22899683

[7] Seong J, Claydon N, Macdonald E, Garner S, Newcombe RG, West N. A randomised clinical trial to determine the abrasive effect of the tongue on human enamel loss with and without a prior erosive challenge. J Dent. 2017;58:48–53. 10.1016/j.jdent.2017.01.011 28161365

[8] Warreth A, Abuhijleh E, Almaghribi MA, Mahwal G, Ashawish A. Tooth surface loss: a review of literature. Saudi Dent J. 2020;32(2):53–60. 10.1016/j.sdentj.2019.09.004 32071532 PMC7016226

[9] Johansson A-K, Omar R, Carlsson GE, Johansson A. Dental erosion and its growing importance in clinical practice: from past to present. Int J Dent. 2012;2012(1):632907. 10.1155/2012/632907 22505907 PMC3312266

[10] Lussi A, Schaffner M. Progression of and risk factors for dental erosion and wedge–shaped defects over a 6–year period. Caries Res. 2000;34(2):182–7. 10.1159/000016587 10773637

[11] Peutzfeldt A, Jaeggi T, Lussi A. Restorative therapy of erosive lesions. Monogr Oral Sci. 2014;25:253–61. 10.1159/000360562 24993273

[12] Belmar da Costa M, Delgado AHS, Pinheiro de Melo T, Amorim T, Mano Azul A. Analysis of laboratory adhesion studies in eroded enamel and dentin: a scoping review. Biomater Investig Dent. 2021;8(1):24-38. 10.1080/26415275.2021.1884558 PMC7889235 33629074

[13] Helfo. Regelverk for tannlege. Helfo; 2024 [cited 2025 Nov 03]. Available from: https://www.helfo.no/tannlege/regelverk-og-takster-for-tannlege/tilstander-som-kan-gi-rett-til-st%C3%B8nad-til-tannbehandling

[14] Helsedirektoratet. Helserefusjonsdata. 2023 [cited 2025 Jul 20]. Available from: https://opne-data.helserefusjon.no/

[15] McKenzie JF, Seabert DM. Encyclopedia of epidemiology. Boslaugh S, editor. Encyclopedia of Epidemiology. Thousand Oaks (CA): Sage Publications; 2008. p. 839–840.

[16] Chen D, Hayes M, Holden A. A global review of the education and career pathways of dental therapists, dental hygienists and oral health therapists. Br Dent J. 2021;230(8):533–8. 10.1038/s41415-021-2836-z 33893429

[17] Oslo Ui. Tannpleie (Bachelor) Universitetet i Oslo. [cited 2025 Nov 03]. Available from: https://www.uio.no/studier/program/tannpleie/

[18] Helsedirektoratet. God klinisk praksis i tannhelsetjenesten. 2011 [cited 2025 Nov 03]. Available from: https://www.helsedirektoratet.no/veiledere/god-klinisk-praksis-i-tannhelsetjenesten/God%20klinisk%20praksis%20i%20tannhelsetjenesten%20%E2%80%93%20Veileder%20(fullversjon).pdf/_/attachment/inline/3a61ee48-164f-423a-ad02-6748ac1479b3:0506b11f2cd7c642750206443eb93ede8c1687ff/God%20klinisk%20praksis%20i%20tannhelsetjenesten%20%E2%80%93%20Veileder%20(fullversjon).pdf

[19] Ariyanayagam Y. A dental hygienist’s and therapist’s guide to the management of tooth erosion. Prim Dent J. 2016;5(3):58–62. 10.1177/205016841600500306 28826465

[20] Tannhelsetjenesteloven. Lov om tannhelsetjenesten: Norwegian Low of Dental Services; 1983 [cited 2025 Jul 20]. Available from: https://lovdata.no/dokument/NL/lov/1983-06-03-54/

[21] NOU:2023:4 MoHaC. Tid for Handling – Personellet i En Bærekraftig Helse- Og Omsorgstjeneste. Helse- og omsorgsdepartementet; 2023 [cited 2025 Jul 20]. Available from: https://www.regjeringen.no/contentassets/337fef958f2148bebd326f0749a1213d/no/pdfs/nou202320230004000dddpdfs.pdf

[22] Breivik A, Kopperud SE, Khan Q, Mulic A, Stein L. Preventive measures and perceived challenges in delivering oral health care for elderly patients: a survey of dental hygienists in Norway. Acta Odontol Scand. 2025;84:1–9. 10.2340/aos.v84.42581 39761069 PMC11758680

[23] Hove LH, Mulic A, Tveit AB, Stenhagen KR, Skaare AB, Espelid I. Registration of dental erosive wear on study models and intra-oral photographs. Eur Arch Paediatr Dent. 2013;14(1):29–34. 10.1007/s40368-012-0004-5 23532811

[24] Mulic A, Árnadòttir IB, Jensdottir T, Kopperud SE. Opinions and treatment decisions for dental erosive wear: a questionnaire survey among Icelandic dentists. Int J Dent. 2018;2018(1):8572371. 10.1155/2018/8572371 30515214 PMC6236703

[25] Lussi A, Jaeggi T, Zero D. The role of diet in the aetiology of dental erosion. Caries Res. 2004;38 Suppl 1:34–44. 10.1159/000074360 14685022

[26] Mulic A, Tveit AB, Wang NJ, Hove LH, Espelid I, Skaare AB. Reliability of two clinical scoring systems for dental erosive wear. Caries Res. 2010;44(3):294–9. 10.1159/000314811 20516691

[27] Helsenorge. Who pays your dental bill? 2025 [cited 2025 Jul 20]. Available from: https://www.helsenorge.no/en/payment-for-health-services/who-pays-your-dental-bill/

[28] Tseveenjav B, Virtanen J, Wang N, Widström E. Working profiles of dental hygienists in public and private practice in Finland and Norway. Int J Dent Hyg. 2009;7(1):17–22. 10.1111/j.1601-5037.2008.00314.x 19215307

[29] Hach M, Aaberg KB, Lempert SM, Danielsen B. Work assignments, delegation of tasks and job satisfaction among Danish dental hygienists. Int J Dent Hyg. 2017;15(3):229–35. 10.1111/idh.12203 28695720

[30] Toven NJWoHV. Tannpleiere i Norge. Yrkesaktivitet og arbeidsforhold. Nor Tannlegeforen Tid 2006. 2006.

[31] Ganss C, Young A, Lussi A. Tooth wear and erosion: methodological issues in epidemiological and public health research and the future research agenda. Community Dent Health. 2011;28(3):191–5. 21916352

[32] Mulic A, Vidnes-Kopperud S, Skaare AB, Tveit AB, Young A. Opinions on dental erosive lesions, knowledge of diagnosis, and treatment strategies among Norwegian dentists: a questionnaire survey. Int J Dent. 2012;2012:716396. 10.1155/2012/716396 22927855 PMC3426243

[33] Michou S, Vannahme C, Ekstrand KR, Benetti AR. Detecting early erosive tooth wear using an intraoral scanner system. J Dent. 2020;100:103445. 10.1016/j.jdent.2020.103445 32750388

[34] Schlenz MA, Schlenz MB, Wöstmann B, Glatt AS, Ganss C. Intraoral scanner-based monitoring of tooth wear in young adults: 24-month results. Clin Oral Investig. 2023;27(6):2775–85. 10.1007/s00784-023-04858-x PMC10264267 36625960

[35] van Nistelrooij N, Maier E, Bronkhorst H, Crins L, Xi T, Loomans BAC, et al Automated monitoring of tooth wear progression using AI on intraoral scans. J Dent. 2024;150:105323. 10.1016/j.jdent.2024.105323 39197530

[36] Witecy C, Ganss C, Wöstmann B, Schlenz M, Schlenz M. Monitoring of erosive tooth wear with intraoral scanners in vitro. Caries Res. 2021;55(3):215–224. 10.1159/000514666 33752205

[37] Mulic A, Tveit AB, Songe D, Sivertsen H, Skaare AB. Dental erosive wear and salivary flow rate in physically active young adults. BMC Oral Health. 2012;12:8. 10.1186/1472-6831-12-8 22443448 PMC3353235

[38] Larsen MJ, Poulsen S, Hansen I. Erosion of the teeth: prevalence and distribution in a group of Danish school children. Eur J Paediatr Dent. 2005;6(1):44–7. 15839833

[39] Milosevic A, Young PJ, Lennon MA. The prevalence of tooth wear in 14-year-old school children in Liverpool. Community Dent Health. 1994;11(2):83–6. 8044716

[40] El Aidi H, Bronkhorst EM, Truin GJ. A longitudinal study of tooth erosion in adolescents. J Dent Res. 2008;87(8):731–5. 10.1177/154405910808700813 18650543

[41] Hasselkvist A, Johansson A, Johansson AK. Dental erosion and soft drink consumption in Swedish children and adolescents and the development of a simplified erosion partial recording system. Swed Dent J. 2010;34(4):187–95. 21306084

[42] Okunseri C, Okunseri E, Gonzalez C, Visotcky A, Szabo A. Erosive tooth wear and consumption of beverages among children in the United States. Caries Res. 2011;45(2):130–5. 10.1159/000324109 21430382

[43] Stenhagen KR, Berntsen I, Odegaard M, Mulic A, Tveit AB. Has the prevalence and severity of dental erosion in Norway changed during the last 30 years? Eur J Paediatr Dent. 2017;18(3):177–82. 29254339 10.23804/ejpd.2017.18.03.02

[44] Almeida e Silva JS, Baratieri LN, Araujo E, Widmer N. Dental erosion: understanding this pervasive condition. J Esthet Restor Dent. 2011;23(4):205–16. 10.1111/j.1708-8240.2011.00451.x 21806751

[45] Marschner F, Kanzow P, Wiegand A. Anamnestic risk factors for erosive tooth wear: systematic review, mapping, and meta-analysis. J Dent. 2024;144:104962. 10.1016/j.jdent.2024.104962 38552999

[46] Helsedirektoratet. Tannhelse-Helsefremmende og forebyggende tiltak for voksne over 20 år 2019 [cited 2025 Jul 20]. Available from: https://www.helsedirektoratet.no/faglige-rad/helsefremmende-og-forebyggende-tannhelsetiltak-for-voksne-over-20-ar

[47] Chan AS, Tran TTK, Hsu YH, Liu SYS, Kroon J. A systematic review of dietary acids and habits on dental erosion in adolescents. Int J Paediatr Dent. 2020;30(6):713–33. 10.1111/ipd.12643 32246790

[48] Bjontegaard MM, Molin M, Kolby M, Torheim LE. Purchase of ultra-processed foods in Norway: a repeated cross-sectional analysis of food sales in 2013 and 2019. Public Health Nutr. 2023;26(9):1743–53. 10.1017/S1368980023001192 37339927 PMC10478042

[49] Helsedirektoratet. Utviklingen i norsk kosthold: Helsedirektoratet; 2024 [cited 2025 May 04]. Available from: https://www.helsedirektoratet.no/rapporter/utviklingen-i-norsk-kosthold-2024

[50] Review WP. Soda consumption by country 2025. World Population Review; 2017 [cited 2025 Jul 04]. Available from: https://worldpopulationreview.com/country-rankings/soda-consumption-by-country

[51] Jarvinen VK, Rytomaa II, Heinonen OP. Risk factors in dental erosion. J Dent Res. 1991;70(6):942–7. 10.1177/00220345910700060601 2045572

[52] Carvalho TS, Colon P, Ganss C, Huysmans MC, Lussi A, Schlueter N, et al. Consensus report of the European Federation of Conservative Dentistry: erosive tooth wear – diagnosis and management. Clin Oral Investig. 2015;19(7):1557–61. 10.1007/s00784-015-1511-7 26121968

[53] Dongo D. Norway’s ban on marketing unhealthy food to children. Food Times; [cited 2025 Jul 04]. Available from: https://www.foodtimes.eu/consumers-and-health/norway-ban-unhealthy-food-marketing-children-2025/

[54] World Health Organization. Global strategy and action plan on oral health 2023–2030. 2024. [cited 2025 Sep 10]. Available from: https://www.who.int/publications/i/item/9789240090538

[55] WHO. Childhood overweight and obesity. [cited 2025 Jul 04]. Available from: https://www.who.int/news-room/questions-and-answers/item/noncommunicable-diseases-childhood-overweight-and-obesity

[56] Botero D, Wolfsdorf JI. Diabetes mellitus in children and adolescents. Arch Med Res. 2005;36(3):281–90. 10.1016/j.arcmed.2004.12.002 15925018

[57] Ludwig DS, Peterson KE, Gortmaker SL. Relation between consumption of sugar-sweetened drinks and childhood obesity: a prospective, observational analysis. Lancet. 2001;357(9255):505–8. 10.1016/S0140-6736(00)04041-1 11229668

[58] Arnadottir IB, Holbrook WP, Eggertsson H, Gudmundsdottir H, Jonsson SH, Gudlaugsson JO, et al. Prevalence of dental erosion in children: a national survey. Community Dent Oral Epidemiol. 2010;38(6):521–6. 10.1111/j.1600-0528.2010.00559.x 20690934

[59] SINTEF. Care4YoungTeeth. [cited 2025 Jul 03]. Available from: https://www.careforyoungteeth.no/#Om-prosjektet

[60] Vejrup K, Engel TN, Lillegard HJ, Strand SK, Stein L, Olsen-Bergem H. Health literacy and health-related habits among conscripts in the Norwegian Armed Forces – a cross-sectional survey. Mil Med. 2025;190(7–8):e1686–92. 10.1093/milmed/usae493 39431798

[61] Norton WE, Funkhouser E, Makhija SK, Gordan VV, Bader JD, Rindal DB, et al. Concordance between clinical practice and published evidence: findings from The National Dental Practice-Based Research Network. J Am Dent Assoc. 2014;145(1):22–31. 10.14219/jada.2013.21 24379327 PMC3881267

